# Cellular Senescence in Acute Liver Injury: What Happens to the Young Liver?

**DOI:** 10.14336/AD.2024.0586

**Published:** 2025-06-10

**Authors:** Keting He, Diwenxin Zhou, Zhangya Pu, Shangci Chen, Yangfan Shen, Shuai Zhao, Xiaohan Qian, Qingqing Hu, Xiaoxin Wu, Zhongyang Xie, Xiaowei Xu

**Affiliations:** State Key Laboratory for Diagnosis and Treatment of Infectious Diseases, National Clinical Research Center for Infectious Diseases, National Medical Center for Infectious Diseases, Collaborative Innovation Center for Diagnosis and Treatment of Infectious Diseases, The First Affiliated Hospital, Zhejiang University School of Medicine, Hangzhou, Zhejiang, China

**Keywords:** cellular senescence, drug-induced liver injury, partial hepatectomy, liver transplantation, radiation-induced liver disease

## Abstract

Cellular senescence, characterized by irreversible cell cycle arrest, not only exists in age-related physiological states, but has been found to exist in various diseases. It plays a crucial role in both physiological and pathological processes and has become a trending topic in global research in recent years. Acute liver injury (ALI) has a high incidence worldwide, and recent studies have shown that hepatic senescence can be induced following ALI. Therefore, we reviewed the significance of cellular senescence in ALI. To minimize the potential confounding effects of aging on cellular senescence and ALI outcomes, we selected studies involving young individuals to identify the characteristics of senescent cells, the value of cellular senescence in liver repair, its regulation mechanisms in ALI, its potential as a biomarker for ALI, the prospect of treatment, and future research directions.

## 1. Introduction

Cellular senescence is an irreversible cell cycle arrest that was first described by Hayflick and Moorhead (1961) [1]. They discovered that normal cultured human fibroblasts irreversibly cease proliferating after approximately 50 divisions, which are known as Hayflick limit. Replicative senescence (RS) was initially attributed to telomere shortening after each division [2]. However, recent evidence suggests that telomere-induced senescence can occur regardless of change in telomere length [3, 4]. In 1997, a study showed that RAS could increase p53, p21, and p16 levels, resulting in cell cycle arrest similar to cellular senescence, known as oncogene-induced senescence (OIS) [5]. Furthermore, various stressors such as DNA damage or impaired DNA repair, epigenetic changes, reactive metabolites, oxidative stress, and mitochondrial dysfunction have also been identified as triggers of cellular senescence [6, 7]. This discovery makes cellular senescence a trending topic and a new therapeutic target for the treatment and prognosis of various diseases.

Cellular senescence has both negative and positive effects. It contributes to aging [8], age-related tissue dysfunction [9], chronic diseases [10], tissue fibrosis [11], tumor promotion [12], immune deficits, and stem cell exhaustion [13]. In contrast, it plays a beneficial role in embryogenesis and development [14], wound and organ repair [13, 15], alleviation of fibrosis [16, 17], and tumor suppression [12].

Acute liver injury (ALI) is a common liver disease worldwide. Viral infections, drugs, toxins, pregnancy, malignant infiltration, autoimmune diseases, Wilson’s disease, Budd-Chiari syndrome can induce ALI [18]. However, the mechanisms underlying the occurrence and development of ALI are complex, and the treatment strategy for liver protection is limited. In some cases, ALI progresses to acute liver failure (ALF), which is a life-threatening critical illness with a mortality rate of approximately 80% [19]. Therefore, new therapeutic targets for ALI are required.

Recent studies have shown that senescence can be induced in epithelial, nonepithelial, and circulating immune cells following ALI, with diverse implications for liver repair. In this review, we elucidate features of cellular senescence, provide an overview of research on its implications in ALI, discuss its significance in liver repair, regulatory mechanisms, therapeutic potential, and raise pertinent questions for future investigations.

Furthermore, aging leads to chronic inflammation and organ dysfunction, and cellular senescence is a common feature of aging [20]. Aging has multiple effects on the liver [21], and ALI is typically more severe in the elderly population [22, 23]. This review focuses on the investigation of ALI in young individuals to identify the factors that contribute to cellular senescence and minimize the potential impact of aging.

## 2. The current markers of cellular senescence

**Figure 1.**
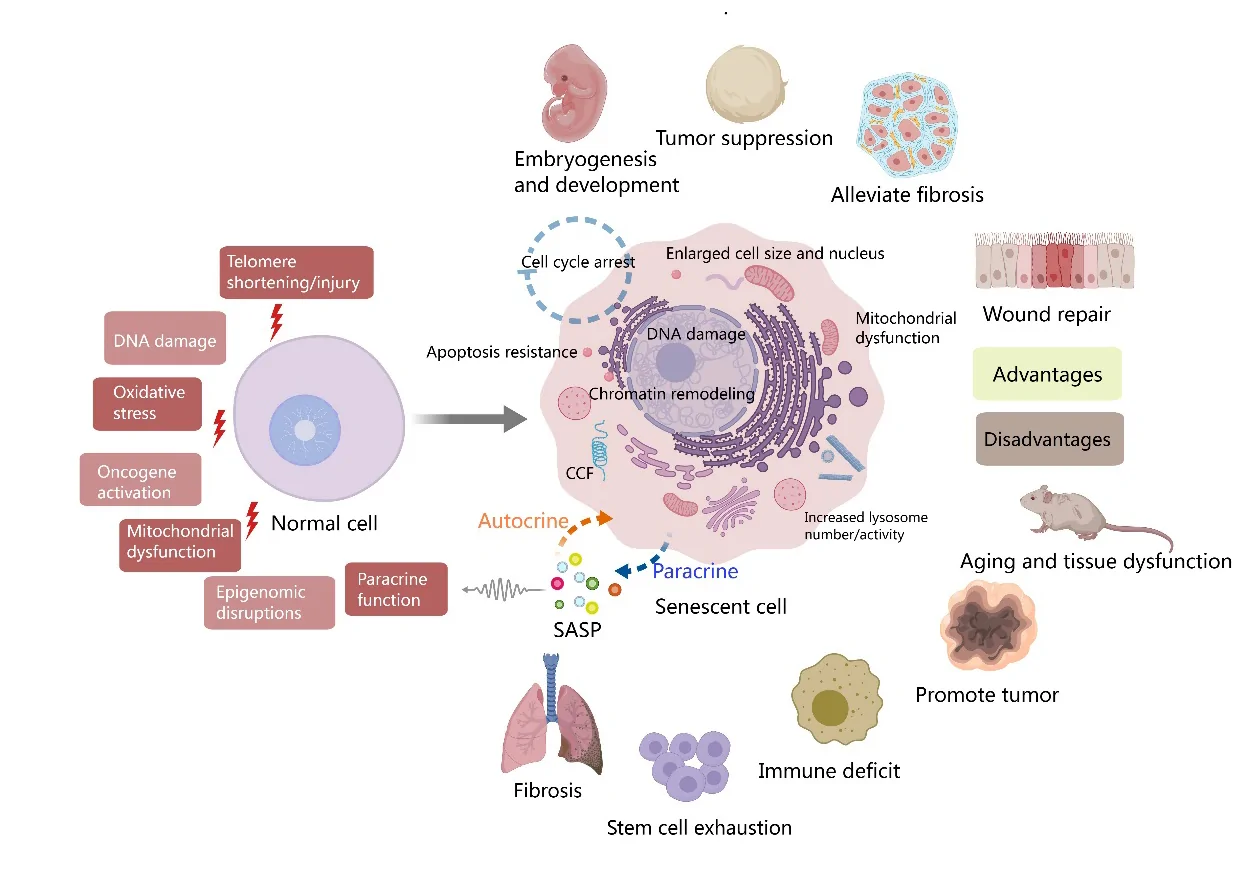
**The causes, features, and functions of cellular senescence**. Normal cells under various stressors will be senescent. Senescent cells often have a large cell size and nucleus, and exhibit cell cycle arrest, DNA damage, chromatin remodeling, cytoplasmic chromatin fragments (CCF), increased lysosome number/activity, and mitochondrial dysfunction. Senescent cells secrete senescence-associated secretory phenotype (SASP), which acts on itself (autocrine) and surrounding cells (paracrine). Its paracrine function can cause paracrine senescence of normal cells. Cellular senescence shows dual effects: it plays a beneficial role in embryogenesis and development, wound repair, alleviating fibrosis, and tumor suppression. Conversely, it contributes to tumor promotion, aging, tissue fibrosis, age-related tissue dysfunction and diseases, immune deficit, and stem cell exhaustion.

Cellular senescence is a complex phenotype that requires a comprehensive approach for its diagnosis. Senescent cells exhibit specific features; therefore, there are various methods to identify in practical applications (Fig. 1).

### 2.1 Morphological changes

Senescent cells are typically enlarged, flat, and multinucleated [24, 25]. Lipofuscin accumulation in senescent cell lysosomes is detectable by staining with Sudan-Black-B [26]. Senescence-associated β-galactosidase (SA-β-gal; detectable at pH 6.0) is commonly used to identify cellular senescence, but not all senescent cell types express SA-β-gal [27]. SA-β-gal activity is not a specific marker for senescence because it indicates increased lysosomal number or activity [28]. In addition, senescent cells typically have an increased number of dysfunctional mitochondria [29]; however, mitochondrial dysfunction is involved in other cellular processes; therefore, it is also not a common marker of senescence [7]. Lamin B1, which is integral to nuclear size, shape, and mechanical decreases in senescent cells, and its overexpression can delay senescence [30, 31].

### 2.2 Cell cycle arrest and apoptosis resistance

Cellular senescence is characterized by cell cycle arrest, which prompts the activation of cyclin-dependent kinase inhibitors (CDKIs), such as p21 (cdkn1a), p16 (cdkn2a), p15 (cdkn2b), and p19 (cdkn2d). However, notably, elevated CDKI levels are also present in non-senescent cells such as macrophages [32], and not all senescent cells express CDKIs [7]. During cellular damage, p53 undergoes phosphorylation and other post-translational modifications, leading to its dissociation from Murine Double Minute 2 (Mdm2). This process activates p21, inhibits the G1/S cell cycle switch, and initiates senescence [33]. P16 maintains cell cycle arrest through the p16-pRB pathway [34]. Furthermore, the expression of proliferation markers, such as Ki-67 and BrdU decrease.

Senescent cells are resistant to apoptosis and promote tissue senescence. This resistance can be attributed to an increase in BCL-2 family proteins, including BCL-2, BCL-W, and BCL-XL [35]. However, this feature is also exhibited by non-senescent cell types, such as tumor cells [36] .

### 2.3. Macromolecular damage

Senescent cells display various types of damaged macromolecules, such as DNA, protein, and lipids [7]. DNA damage is commonly used to detect senescence. Numerous exogenous and endogenous stressors can induce DNA damage, which is detectable through the formation of DNA damage foci containing phosphorylated histone H2A.X (γH2A.X). The subsequent outcomes depend on DNA damage response (DDR), which is crucial for detecting and repairing DNA damage. DDR is regulated by ataxia telangiectasia mutated (ATM), ATM and Rad3-related (ATR), and DNA-dependent protein kinases (DNA-PK) [37, 38].

Chromatin remodeling is important in this process. A specific type of heterochromatin called senescence-associated heterochromatic foci (SAHF), which has high DNA density and a low gene transcription rate, accumulates in senescent cells. Histone markers (H3K9me3, H3K27me3, and H3K23me2) can be used to identify SAHF [39, 40]. Senescent cells release nuclear chromatin fragments into the cytoplasm, forming cytoplasmic chromatin fragments (CCF) [41]. CCF formation is closely related to DDR and DNA repair and regulates the senescence-associated secretory phenotype (SASP) [42].

### 2.4. SASP

Senescent cells have an active metabolism, and produce SASP, which is a complex mixture of proinflammatory cytokines, immunomodulatory cytokines, and chemokines [43]. Its composition varies depending on the factors inducing senescence [44]. SASP is dynamically and spatially regulated, and the beneficial or harmful effects of senescence can vary depending on SASP composition [45].

Identifying senescence is a complex and confusing process due to the lack of specificity of the individual biomarkers. Thus, combining different features of senescent cells may increase our confidence in identifying them. However, there are variations among the different types of senescent cells. In addition, it is important to distinguish between quiescence and cellular senescence. Quiescence is reversible cell cycle arrest; when quiescent cells receive certain stimulus, they will activate again. Although quiescent cells have some similarities with senescent cells, they have unique characteristics, such as cell cycle arrest at the G0 phase, increased levels of p21, p27, and p57, decreased metabolic activity, energy production, and biosynthesis, and alterations in cellular structures [46]. To address these challenges, some studies have used high-throughput technologies, such as single-cell RNA-sequencing, to investigate senescence heterogeneity and uncover novel senescence biomarkers [47-50]

## 3. Cellular senescence in acute liver injuries (ALIs)

### 3.1 Cellular senescence in epithelial cells

Hepatocyte senescence is common in various ALIs, such as partial hepatectomy (PH) [51], viral hepatitis [52], drug-induced liver injury (DILI) [52, 53], radiation-induced liver disease (RILD) [54], hereditary tyrosinemia (HT) [55], and liver ischemia-reperfusion injury (IRI) [56]. Cholangiocyte senescence has been observed in chronic biliary liver disorders such as biliary atresia (BA), primary biliary cholangitis (PBC), primary sclerosing cholangitis (PSC), and chronic parenchymal liver diseases [57-60]. However, this phenomenon has seldom been observed in patients with ALI. Several studies have shown that cholangiocyte senescence occurs in murine models that replicate liver procurement and static cold storage [61], as well as in human liver transplant biopsies that exhibit acute allograft rejection [62, 63].

### 3.2 Cellular senescence in nonepithelial cells and circulating cells

Liver sinusoidal endothelial cells (LSECs) are specialized endothelial cells located between the blood and liver cells. They are essential for maintaining hepatic homeostasis [64]. LSECs can acquire vasoconstrictor, proinflammatory, and prothrombotic properties during liver injuries [65]. Although LSEC senescence has been demonstrated in mice subjected to PH [66], its manifestation in humans remains unexplored. Similarly, the senescence of hepatic stellate cells (HSCs), which has been documented in liver fibrosis and cancer of different etiologies [17, 67-69], has rarely been studied in ALI. A recent study reported HSC senescence in young (2-3 months old) mice subjected to PH and revealed its function in liver regeneration [70].

**Figure 2.**
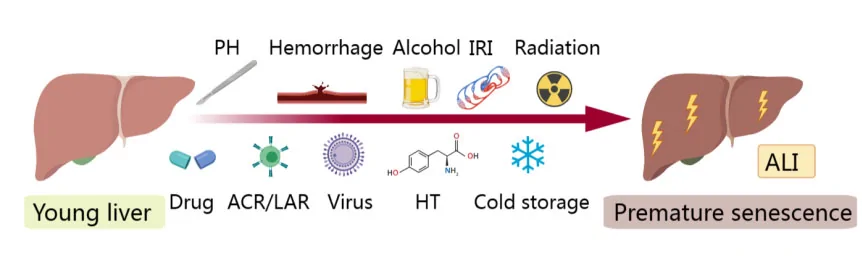
**Acute liver injuries induce premature senescence of the liver**. Many causes of ALI, including drug, PH, alcohol, cold storage, virus, hemorrhagic shock, radiation, acute cellular rejection (ACR/LAR), hereditary tyrosinemia (HT), and ischemia-reperfusion injury (IRI) can induce premature senescence of the young liver.

A recent study using single-cell RNA sequencing has revealed the senescence of peripheral blood mononuclear cells (PBMCs) in patients with acute alcohol-associated hepatitis (AH). This study is unique because it demonstrated immune cell or extrahepatic cellular senescence in ALI [71] (Fig. 2).

## 4. The roles of cellular senescence in ALI

### 4.1 Promote regeneration and inhibit carcinogenesis

Some studies have shown that cellular senescence promoted liver regeneration directly or indirectly. In a PH model, eliminating senescent cells via different methods would impair liver weight recovery [15, 70]. Senescent HSCs secrete IL-6 and CXCR2 ligands, which are components of the SASP. IL-6 activates downstream pathways, including STAT3 and YAP, and synergizes with CXCL2 to activate the ERK1/2 pathway, thereby promoting hepatocyte proliferation [70]. In addition, hepatic senescence creates favorable conditions for hepatocyte transplantation. Pretreatment of a recipient's liver with radiotherapy before hepatocyte transplantation can induce senescence in the recipients’ cells and enhance engraftment of transplanted hepatocytes, leading to extensive repopulation of the liver. Radiation impedes cell proliferation, which indirectly provides a growth advantage to transplanted (healthy) hepatocytes over host (diseased) hepatocytes [72]. The results indicate the positive role of senescence in promoting liver regeneration.

In addition, hepatic senescence can inhibit carcinogenesis. In a fumarylacetoacetate hydrolase (FAH(-/-)) model, severe acute liver injury (SALI) induces hepatocyte senescence and activates macrophages. Furthermore, cellular senescence induced by SALI inhibits carcinogenesis by activating immune surveillance initiated by macrophages [55].

### 4.2. Impair liver regeneration

**Figure 3.**
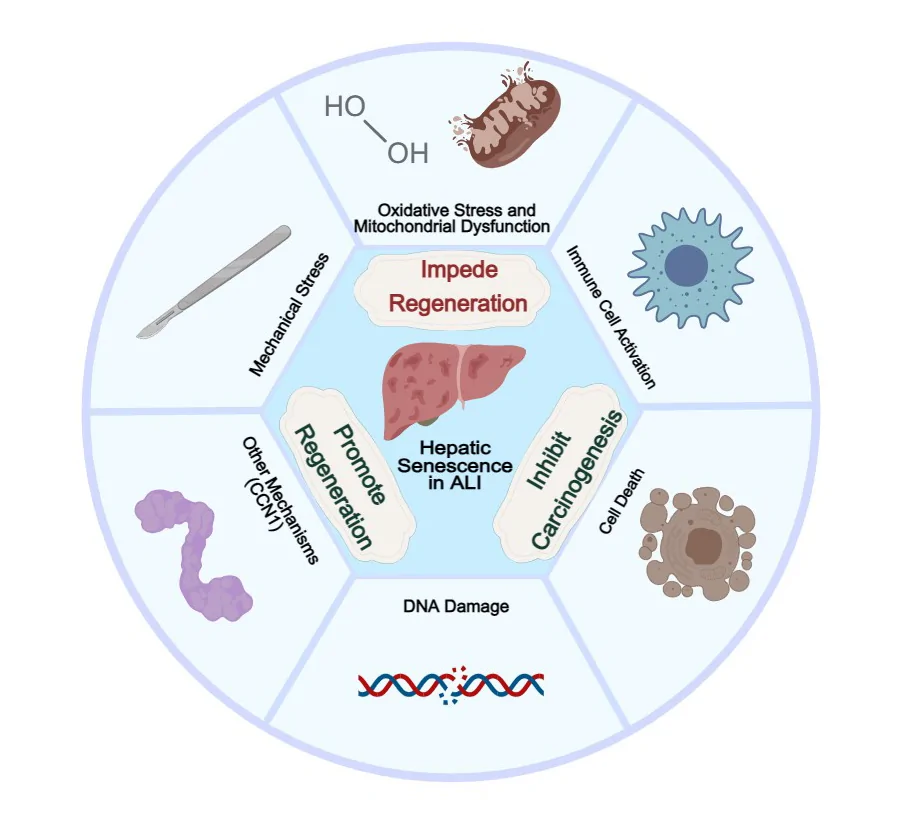
**The mechanisms and significance of hepatic senescence in ALI**. The mechanisms of hepatic senescence in ALI can be divided into several types including “oxidative stress and mitochondrial dysfunction”, “immune cell activation”, “DNA damage”, “cell death”, “mechanical stress”, and “other mechanisms”. Cellular senescence following ALI can impede liver regeneration, promote liver regeneration, and inhibit carcinogenesis.

Based on current research, hepatic senescence exhibits more disadvantages than advantages. First, animals with p21-knockout or p53-knockout often show reduced hepatic senescence and reduced liver injury [15, 52, 61, 73, 74]. Second, eliminating senescent cells has been proven to reduce liver injury, promote liver regeneration, and preserve biliary tract architecture [61, 75]. Furthermore, inhibiting paracrine senescence, SASP, or regulating some senescence-related signal pathways can decrease liver injury or promote liver recovery [52, 53, 56, 66, 76-80]. The specific mechanisms are described below (Fig. 3).

## 5. Mechanism of cellular senescence in ALI

There are various factors contributing to hepatic senescence, but the basic mechanism is thought to be the regulation of the p53/p21 and Rb/p16 axes [81]. Following PH, p21 and p19 levels increase, and p16 levels decrease. Hepatocytes do not proliferate in animals with p21 overexpression or p16 knockout who underwent PH. Conversely, elimination of p21 promotes hepatocyte progression through the G1 phase, facilitating liver regeneration [82-85]. The changes in p21 and senescence are regulated by the p53/MDM2 axis [84, 86]. A similar phenomenon has also been observed in various ALI models, such as DILI, RILI, HI, and FAH(-/-) knockout [15, 52, 73, 74]. Based on the literature, we classified the mechanisms of cellular senescence into several categories (Table 1).

### 5.1. Oxidative stress and mitochondrial dysfunction promote cellular senescence in ALI

Several animal models have proven that hepatic senescence in ALI is induced by oxidative stress and mitochondrial dysfunction [53, 56, 76]. Mitochondrial oxidative stress is considered the predominant cellular event in (acetaminophen) APAP-induced liver injury [87]. In the case of APAP overdose, excess of N-acetyl-p-benzoquinone imine (NAPQI) is produced, which reduces glutathione (GSH) levels. This results in mitochondrial dysfunction and oxidative stress [88, 89]. Dysfunctional mitochondria produce ROS and contribute to the formation of CCF and SASP in senescent cells through JNK. This process is inhibited by 53BP1 [76]. In CCl4-induced ALI, neutrophils can produce ROS which expedites telomere shortening in neighboring cells, causing premature senescence. This process requires direct cell-to-cell contact [53].

IRI typically results in the release of ROS [90]. Glutathione peroxidase 3 (GPx3) is an antioxidant that helps resist excessive oxidative stress [91]. In small grafts, liver injury after ischemia-reperfusion is severe, with lower GPx3 levels and a higher proportion of senescent hepatocytes. Low GPx3 levels accelerate hepatic senescence in a liver transplantation model. CD44, Nox4, SERPINB2, and IFNG are considerably upregulated during cellular senescence, suggesting their potential involvement in oxidative stress-induced hepatic senescence [56].

**Table 1:** The type of senescent cells and mechanism.

ALI	Evidence resources	Senescent cells	Senescence markers that are used	Mechanism	Associated therapies improving ALI	Ref.	Year
**Fulminant hepatitis**	Human (aged 3 years or less)	Unknown	p21, p16, p53, SA-β-gal	NA	NA	[126]	2010
**ALF**	Human (under 18 years of age)	Hepatocytes	p21 and p53	NA	NA	[127]	2024
**Virus, drug, and cryptogenic hepatitis**	Human	Hepatocytes (mainly)and non-parenchymal cells Hepatocytes (mainly)and non-parenchymal cells	p21, 16, SA-βGal, and Ki-67	NA	NA	[52]	2018
**DILI**	APAP (human)		p21, DcR2, γH2Ax,and SA-βGal	NA	NA	[52]	2018
	CCl4 and APAP (male mice, 8w)		p21, p16, SA-βGal, BrdU or Ki67, γH2Ax, SAHF, and SASP	Macrophage-derived TGF β1 can mediate senescent hepatocytes spreding to neighboring hepatocytes, causing paracrine senescence.	TGFβR1 inhibitors AZ12601011 or SB525334	[52]	2018
	IMR90 primary human fibroblasts/APAP (male mice, 9w)	Unknown	CCF, γH2Ax, SASP, H4K16ac, H4, H3K9ac, and H3	Mitochondria-ROS-JNK signaling pathway drives CCF formation and hence the SASP, and 53BP1 linked to JNK is the inhibitor of this process.*	TSA (HDACi)	[76]	2020
	Human MRC5 fibroblasts/human liver/CCl4 (male mice, 8-10w)	Hepatocytes	TAF, p21, p16, SADS, hepatocyte nuclear size, SADS, Lamin B1, and SASP	Neutrophils produce ROS to accelerate telomere shortening in neighboring cells, causing premature senescence depending on direct cell-to-cell contact.	Neutralizing antibody against Ly6G	[53]	2021
	CCl4 (male mice, 8w)	Hepatocytes	P21, p16, SA-βGal, and SASP	NA	ASCs derived exosomes decorated with vitamin A and quercetin	[77]	2021
**PH**	2/3 PH (male mice, 2-3m)	HSCs (peak on day 2)	p16, SASP, Ki67, and γH2Ax	CCN1 stimulates HSC senescence through direct binding to integrin α6β1.	Recombinant IL-6, recombinant CXCL2 or combination	[70]	2022
	PH (male mice,6-8w)	LSECs (87%), hepatocytes (7%) (peak on day 14)	p21, p16, p53, Pai1, IGFBP3, Gata4, SASP, Ki67, and SA-βGal	The activation of Notch inhibits Sirt1 to inducing LSEC senescence. Incomplete remodeling of liver sinusoids affects shear stress and cause (eNOS) signaling inactivation, leading to senescence.	SRT1720 (Sirt1 agonists)	[66]	2022
	70% PH(male and female mice, 6w)	Hepatocytes	p21, cyclin A/B/D/E, SASP, SA-βGal and Brud	Autophagy deficiency induces hepatocyte senescence and impair regeneration. Activation of autophagy maintains healthy mitochondria and stimulate mitochondrial metabolism.	NA	[51]	2014
**Liver procurement and static cold storage**	Human/mice (male and female, 8-12w)	Cholangiocytes	Ki-67, PCNA, γH2Ax, SASP, senescence inductors, cell cycle, and SA-β-gal	DCR2 is upregulated in senescent cholangiocytes to resist apoptosis and to maintain the senescent phenotype.	D+Q/ ABT-737	[61]	2022
**IRI/ liver transplatation**	Mice (male, 6-8w)/ rats (male, 300-350g)	Hepatocytes	p16, SA-β-Gal, and ki-67	IRI will stimulate GPx3 expression. GPx3 is an antioxidant to resist excessive oxidative stress. Small-for-size liver graft show lower production of GPx3 and increased senescence.	GPx3 delivered by hiPSC-MSCs	[56]	2018
**ACR**	Human	Cholangiocytes	p21, ki-67, and γH2Ax	NA	NA	[62]	2013
	Human	Cholangiocytes	p21, p16, and p53	NA	NA	[63]	2022
**RILD**	Rat (200-250g) A single dose of 25 Gy	Hepatocytes	p16, p21, cdk1 SA-β-Gal cell size, and SASP	NA	NA	[54]	2014
	Mice (female, 8-12w)8 Gy total body irradiation	Unknown	γH2Ax, 53BP1, and BrdU	The clearance/accumulation of senescence is independent of T, B or NK cells functions as well as p53.	NA	[94]	2010
**AH**	Human	PBMCs	telomere length	Telomere maintaining molecules including shelterin and telomerase may contribute to the change of telomere length.	NA	[71]	2023
**HI**	Rats (male, 10-12w)	Unknown	p21, p16, p27, P53, MDM2, p-P53, p-MDM2, cyclin D1, cdk2/4/6, SASP and SA-β-gal	P21 and p27, but not p16, may trigger senescence following HI.	NA	[15]	2020
**HT**	Fah-/- (mice)	Hepatocytes	cell size, P21, P53, SAHF, SASP and SA-β-gal	Hepatocyte senescence is strongly induced in SALI rather than MCLI.	NA	[55]	2018

NA: the article did not explore the mechanism or did not use the drug

* The mechanism is from IMR90 primary human fibroblasts, but the results indicate that mitochondria are required for the formation of CCFs and hence the SASP program.

ACR: Acute Cellular Rejection; AH: Alcoholic Hepatitis; ALI: Acute Liver Injury; APAP: Acetaminophen; ASCs: Adipose mesenchymal stem cells: CCl4: Carbon Tetrachloride; CCF: Cytoplasmic chromatin fragments; DILI: Drug-Induced Liver Injury; HI: Hemorrhagic Shock Injury; HSCs: hepatic stellate cells; HT: Hereditary Tyrosinemia; HDACi: Histone deacetylase inhibitors; IRI:Ischemia-Reperfusion Injury; LSECs:Liver Sinusoidal Endothelial Cells; MSCs:Mesenchymal stem cells; HiPSC-MSCs:MSCs derived from human induced pluripotent stem cells; PH:Partial Hepatectomy; RILD:Radiation-Induced Liver Disease; SA-β-gal: Senescence-Associated β-Galactosidase; SADS: senescence-associated distension of satellites; SAHF: Senescence-Associated Heterochromatic Foci; SASP: Senescence-Associated Secretory Phenotype; TAF: Telomere Dysfunction-Associated Foci

### 5.2 Immune cell activation promotes cellular senescence in ALI

In ALI, immune cells accumulate in the liver [92, 93]. In DILI, hepatocytes that undergo genetic manipulation, such as Mdm2 deletion, exhibit cell-autonomous senescence. They propagate senescence to neighboring hepatocytes, inducing non-cell-autonomous senescence. This phenomenon, known as paracrine senescence, relies on macrophage-derived transforming growth factor beta 1 (TGFβ1). Myeloid specific *TGFβ1* deletion could cause equivalent ALI but improved liver regeneration. Inhibiting leukocyte recruitment in the Δ*Mdm2*^Hep^ model could also reduce p21 expression and could improve hepatocyte regeneration [52].

Neutrophils are another factor that induces liver senescence in ALI. Neutrophils in the peripheral blood of healthy individuals can cause telomere dysfunction and senescence in precision-cut liver slices (PCLS). In ALI induced by high doses of CCl4, there is an increase in neutrophil infiltration, telomere dysfunction-associated foci (TAF) count, TAF-positive hepatocytes, p21-positive hepatocytes, p16 expression, senescence-associated distension of satellites (SADS), and SASP [53, 77]. However, eliminating senescent cells would not induce notable differences in the number of macrophages and neutrophils in a liver after PH [70]. These results suggest a complex causal relationship between hepatic senescence and immune cells.

Macrophages can also clear senescent cells under pathological conditions. A previous study exhausted macrophages in a PH model and found that this promoted LSEC senescence and inhibited cell proliferation, indicating their dual effects [66]. Furthermore, to explore the relationship of cellular senescence and other immune cells in ALI, another study investigated the function of the immune system in senescence clearance/accumulation in Rag2^-/-^ and γc^-/-^mice. The results showed that senescence clearance/accumulation is independent of T, B, and NK cell functions, despite their critical roles in clearing damaged cells and orchestrating DNA damage responses and cell fate [94].

### 5.3. DNA damage promotes cellular senescence in ALI

DNA replication and genome stability are essential for organisms [95]. Deficiency of DNA methyltransferase 1 (DNMT1), excision repair cross-complementation group 1 (Ercc1), or telomerase contributes to senescence in the livers of young mice, impairing liver regeneration after PH. These studies revealed a resemblance between accelerated senescence resulting from a DNA repair defect and the natural aging process, highlighting the protective functions of genome stability in mitigating liver injuries [78-80]. A single sublethal dose of total-body irradiation can cause a sharp increase in 53BP1 foci, indicating DNA damage within 24 h. Moreover, p16 expression was observed within 12 weeks in a mouse liver. Although 53BP1-positive cells declined spontaneously, their elevation persisted for at least 45 weeks compared with that in the controls [94]. Compared to heavy drinkers without liver disease and healthy controls, the PBMCs of patients with AH exhibited enrichment of senescence pathways and differential expression of genes associated with telomere maintenance and DNA damage repair pathways [71]. However, the mechanism of PBMC senescence remains unclear.

### 5.4. Cell death and cellular senescence in ALI

Cell death can clear damaged, infected, or obsolete cells, which is required for the survival and fitness of organisms [96]. Apoptosis is a programmed cell death that removes injured cells in an orderly and efficient way to maintain normal metabolic activity [97]. Cold storage can cause significant damage to biliary architecture. Longer cold storage periods have been linked to reduced cholangiocyte proliferation and increased cholangiocyte senescence. Hepatocytes upregulated the expression of apoptosis-related genes, while senescent cholangiocytes exhibit a high expression of Decoy receptor 2 (DCR2), a factor that can resist apoptosis [98, 99]. *In vitro* studies have shown that CRISPR-mediated DCR2 knockdown increases cholangiocyte proliferation and reduces senescence but has the opposite effect in hepatocytes. Interestingly, although injuries in livers obtained from p21-knockout mice still occur after cold storage, regenerative capacities of the biliary tract are retained, suggesting the importance of senescence in the development of biliary injury [61].

Autophagy is a process by which cytoplasmic material enters the lysosome and undergoes degradation. This process increases during regeneration. Loss of liver-specific autophagy-related gene 5 (Atg5) and autophagy induces hepatocyte senescence and impairs regeneration. Therefore, autophagy plays an important role in promoting regeneration and preventing hepatocyte senescence [51].

### 5.5. Mechanical stress promotes cellular senescence in ALI

Incomplete remodeling of liver sinusoids after PH affects shear stress and inactivates endothelial nitric oxide synthase (eNOS) signaling, leading to LSEC senescence. Activation of this pathway can reduce LSEC senescence. The Notch signaling pathway regulates shear stress, and its activation can inhibit sirtuin 1 (Sirt1) transcription, accelerating LSEC senescence and impeding liver regeneration. In addition, the study found that cellular senescence peaked 14 days after PH (the late stage of PH) in mice, with senescence mainly detected in LSECs [66].

### 5.6 Other mechanisms

Other mechanisms may promote cellular senescence in the liver. Within 2 days after a two-third PH, most (87%) senescent cells were HSCs induced by elevated central communication network factor 1 (CCN1). Furthermore, HSCs comprise most senescent cells during this early stage, with only 7% being hepatocytes [70]. Several studies have reported hepatocyte senescence in patients with chronic alcoholic hepatitis, but limited evidence exists regarding cellular senescence in the liver in acute alcoholic injuries. However, downregulation of YAP and upregulation of NFATc4, ZNF281, and CCN1 have been shown to trigger the senescent phenotype in alcohol-treated hepatocytes [100-102].

## 6. Therapeutic potentials of targeting cellular senescence in ALI

Senotherapies targeting cellular senescence have been extensively studied in the context of aging-related dysfunctions and chronic diseases. Identifying senescence in liver cells and its involvement in ALI suggests the therapeutic potential of drugs that target senescence and may reveal new avenues for ALI treatment. Senotherapy can be classified into two categories based on its mechanisms: senolytic and senomorphic. Other therapies that target senescence have also been explored. This review discusses their role in ALI (Table 2).

### 6.1 Senolytics

Senolytics are drugs that can eliminate senescent cells or induce senolysis, including BCL-2 family inhibitors, HSP90 inhibitors, p53 modulators, natural products and their analogs, cardiac glycosides, galactose-modified prodrugs, proteolysis-targeting chimera [6, 103]. Dasatinib plus quercetin (D+Q) is a common senolytic cocktail that selectively eliminates senescent cells. Studies have demonstrated its efficacy in improving physical function and increasing lifespan in old mice [104, 105]. Clinical trials investigating D+Q have shown its potential to alleviate physical dysfunction in patients with idiopathic pulmonary fibrosis [106], and reduce senescent cell burden in patients with diabetic kidney disease [107]. BCL-2 family inhibitors such as ABT-737 and ABT-263 (Navitoclax) can also increase median survival of progeroid mice [108]. They have been used a treatment for many diseases [109-111].

**Table 2:** Therapies targeting senescence in ALI.

Type of the drug	Drug	ALI models	Results	Mechanisms	Ref.	Year
Senolytics	D+Q	HI (male rats, 10-12w)	Increase mortality	Eliminate senescent cells may have off-target effects.	[15]	2018
	ABT-263	PH (mice, 2-3m)	Impair liver regeneration		[70]	2018
	ABT-737	IRI (male and female mice, 8-12w)	Reduce liver injury		[61]	2022
	D+Q/ ABT-737	Discarded human donor liver/smurine models that recapitulate liver procurement and static cold storage (male and female mice, 8-12w)	Biliary tract architecture is better preserved during cold storage		[61]	2022
	ABT-263/D+Q	RILD (male mice, 5m)	Reduce liver injury		[75]	2022
Senomorphics/anti-SASP activity	Metformin	RILD (male mice, 5m)	Reduce liver injury	Inhibiting SASP via diminishing NOX4 activity in senescent cells.	[75]	2022
	low dose TSA (HDACi)	APAP (male mice, 9w)	Suppress inflammation	Suppresses down-regulation of nuclear-encoded mitochondrial oxidative phosphorylation genes, up-regulation of NRF2 target genes, oxidative damage, CCFs, inflammation, and secondary senescence.	[76]	2020
**SASP**	Recombinant IL-6, recombinant CXCL2 or combination	2/3 PH (male mice, 2-3m)	Promote liver regeneration	Promote hepatocyte proliferation via stimulating YAP, STAT3, and ERK1/2 activation.	[70]	2022
**Drugs that block paracrine senescence**	TGFβR1 inhibitors AZ12601011 or SB525334	CCl4 and APAP (male mice, 8w)	Reduce liver injury and mortality	Reduce local TGF-β pathway activation in perinecrotic hepatocytes.	[52]	2018
	Neutralizing antibody against Ly6G	CCl4 (male mice, 8-10w)	Decrease telomere dysfunction and senescence-associated markers, and increase compensatory proliferation of hepatocytes	Inhibit neutrophil infiltration in the liver.	[53]	2021
**Cell therapies combined with drugs**	GPx3 delivered by hiPSC-MSCs	IRI (male mice, 6-8w; rats, 300-350g)	Reduce liver injury	Down-regulate CD44, Nox4, IFNG, and SERPINB2. CD44, Nox4, SERPINB2 may be related to initiation of oxidative stress induced cellular senescence. Suppresse hepatic senescence and apoptosis.	[56]	2018
	ASCs derived exosomes decorated with vitamin A and quercetin	CCl4 (male mice, 8w)	Reduce liver injury	Reduce the levels of p21, p16, and SASPs, and the positive rate of SA-β-gal staining.	[77]	2021
**Others**	SRT1720 (Sirt1 agonists)	PH (male mice,6-8w)	Promote liver regeneration and improve sinusoid remodeling	Neutralize the up-regulation of P53, P21, and P16 caused by Notch activation and eliminate Notch-driven LSEC senescence.	[66]	2022

ALI: Acute Liver Injury; APAP: Acetaminophen;ASCs: Adipose mesenchymal stem cells; CCl4: Carbon Tetrachloride;CCF: Cytoplasmic chromatin fragments; DILI: Drug-Induced Liver Injury; D+Q: Dasatinib plus quercetin; HI: Hemorrhagic Shock Injury; HSCs: hepatic stellate cells; HDACi: Histone deacetylase inhibitors; IRI:Ischemia-Reperfusion Injury; LSECs:Liver Sinusoidal Endothelial Cells; MSCs:Mesenchymal stem cells; HiPSC-MSCs:MSCs derived from human induced pluripotent stem cells; PH:Partial Hepatectomy; RILD:Radiation-Induced Liver Disease; SASP: Senescence-Associated Secretory Phenotype; TSA:Trichostatin A

Several studies have investigated whether senolytics can improve liver repair in young individuals with senescence-associated ALI. However, these results were contradictory. In HI rat models, D+Q treatment increased mortality. To investigate the role of cellular senescence in HI, niacin, dichloroacetate, and resveratrol (NiDaR), a drug combination that improves organ function and survival after HI was administered to HI rats and senescence-related markers in the liver were measured. The results showed that NiDaR did not suppress cellular senescence [15]. Another study showed that ABT-263 reduced the rate of remnant liver recovery in young mice (2-3 months old) after PH [70].

In contrast, ABT-737 accelerated the recovery of liver mass, improved histological changes, and reduced the elevation of liver enzymes after PH in adult mice (6-8 months old). Although ABT-737 is believed to function as a senolytic, it primarily decreases the levels of p21 and SASP genes in the early stages after a PH, with no effect on p16 expression [84]. In addition, ABT-737 reduces IRI and promotes hepatocyte proliferation [61]. ABT-263 or D+Q intervention may improve RILD and reduce senescent hepatocyte frequencies in 5-month-old mice; however, this has not been tested in younger mice [75]. Furthermore, using D+Q or ABT-737 before liver procurement and cold storage may reduce DCR2 abundance, biliary damage, cholangiocyte senescence, and hepatocellular death, and may improve biliary regeneration. Continuous perfusion of D+Q into hepatic segments after dissection can improve the overall preservation ratio and alleviate biliary injury. Therefore, senolytics may be beneficial for the liver before transplantation [61].

In summary, senolytics have both positive and negative effects on ALI treatment according to the currently available literature; thus, precautions should be taken when using these drugs.

### 6.2 Senomorphics (anti-SASP) and SASP

Senomorphics are drugs that suppress SASP without eliminating senescent cells. These drugs include rapamycin, metformin, aspirin, NF-κB inhibitors, p38MAPK inhibitors, JAK/STAT inhibitors, ATM inhibitors, statins, and natural products [103]. Although numerous drugs have been identified to improve liver repair after ALI through various pathways [112-118], it remains unknown whether they can exert therapeutic effects by influencing SASP.

Metformin improves RILD and senescence markers in the liver of 5-month-old mice by inhibiting SASP and reducing NADPH oxidase 4 (NOX4) activity in senescent cells [75]. Trichostatin A (TSA), a histone deacetylase (HDAC) inhibitor, can improve mitochondrial function and suppress oxidative stress, CCFs, and SASP in the APAP model at low doses [76]. High doses of HDAC inhibitors have been reported to eliminate senescent cells [119]. Therefore, TSA exhibits anti-SASP activity at low doses and senolytic activity at higher doses [76].

Although SASP has negative aspects, it plays a positive role in promoting regeneration. After PH, senescent HSCs secrete IL-6 and CXCR2 ligands as part of the SASP. ABT-263 eliminates senescent cells and impedes liver regeneration. However, the administration of recombinant IL-6 or CXCL2 can restore regenerative conditions. The combination of IL-6 and CXCL2 further strengthens this effect [70]. Although the study did not show whether IL-6 or CXCL2 could enhance regeneration in mice subjected solely to PH (no ABT-263 pretreatment), it suggests the possibility that the SASP could be used as a therapeutic agent for treating ALI.

### 6.3 Other therapies

The potential therapeutic value of related pathways in the treatment of ALI was demonstrated by the association between immune cells and hepatic senescence. Studies have explored whether blocking TGF-β production by macrophages or ROS production by neutrophils, which can induce paracrine senescence of the liver, can alleviate ALI. AZ12601011 and SB525334, inhibitors of TGFβR1, can reduce hepatocyte senescence and liver injury and promote hepatocyte proliferation even when administered 12 h after APAP exposure. In contrast, N-acetylcysteine, a traditional treatment for APAP poisoning, is only effective when administered within several hours of exposure [52]. Pretreatment of mice with a neutrophil-neutralizing antibody against Ly6G can deplete neutrophils, decrease telomere dysfunction and senescence-associated markers, and increase compensatory proliferation of hepatocytes [53]. Previous studies have focused on DILI, and it is unclear whether the identified drugs can be applied to other types of ALI. Nevertheless, these studies have emphasized the significant role of immune cells in liver senescence in young individuals.

Cellular therapies are promising approaches for targeting cellular senescence in ALI. Inhibiting hepatic senescence is possible by delivering GPx3 through MSCs derived from human induced pluripotent stem cells (hiPSC-MSCs). The suppressive effect of GPx3 on hepatic senescence is attributed to four candidate genes (*CD44*, *Nox4*, *IFNG*, and *SERPINB2*) [56]. Exosomes derived from adipose mesenchymal stem cells (ASCs) decorated with vitamin A and quercetin have been shown to improve the stability and bioavailability of quercetin and alleviate CCL4-induced ALI and hepatocyte senescence [77].

Sirtuins are a family of nicotine-adenine dinucleotide (NAD)-dependent deacetylases with multiple functions. Studies have shown that sirtuins have antiaging and therapeutic properties [120-122]. SRT1720, a Sirt1 activator, has been found to reduce LSEC senescence, facilitate sinusoid remodeling, increase hepatocyte proliferation, and promote liver repair after PH [66].

## 7. Cellular senescence as a prognosis biomarker in ALI

Cellular senescence has exhibited potential in predicting prognosis of chronic liver diseases and liver cancers [123-125]. Some studies explored the relationship between senescence and ALI prognosis.

In human specimens (including children with end-stage liver disease), the severity of ALI is positively correlated with the extent of cellular senescence, and greater number of senescent hepatocytes are associated with a poor outcome (patients either died or underwent liver transplantation) [52, 126, 127]. After liver transplantation, 15-25% of recipients experience acute cellular rejection (ACR) within 1-3 months. Late acute cellular rejection (LAR) occurs after the first three months. It was reported that T cell-mediated acute rejection is positively associated with the number of senescent cholangiocytes [62]. Compared to ACR, LAR exhibits more senescent cells and is more prone to progression to chronic rejection [63]. These findings suggest that bile duct senescence may exacerbate immunological damage, foster therapy resistance, and predict a poor prognosis. For patients with AH, although there is no difference in telomere length between heavy drinkers without liver disease and healthy controls, based on the Z-score cutoff, lower telomere length in AH is associated with higher patient mortality [71]. However, these studies are limited, exploring the potential of senescence markers as new biomarkers for ALI is a valuable topic of discussion.

## 8. Questions and future directions

Although cellular senescence in ALI has received increased attention in recent years, numerous questions require further investigation.

Although ALI is common worldwide, its relationship with cellular senescence has not been well studied. Whether the mechanisms described above can be extrapolated to all ALI types in patients of all ages remain unclear. Which types of cells undergo senescence in ALI? And do senescent cells in ALI process heterogeneity? New techniques such as single-cell sequencing may be helpful in answering these questions. Senescence is a dynamic process, and the composition of senescent cells and the SASP may change continually during different phases of ALI [66, 70]. However, these observations were based on several independent studies, and there is a lack of comprehensive evidence to fully understand cellular senescence at different stages of ALI.

Senescence can promote liver repair, but it can also worsen damage. Several studies have shown that senescence peaks after ALI and gradually decreases [66, 70], indicating that the liver may spontaneously clear senescent cells. The aggressive elimination of senescent cells may be counterproductive. Therefore, distinguishing between the nature and role of senescence in ALI, whether intervention is required, and how and when to intervene requires further exploration.

Furthermore, although many researchers have investigated senolytics and senomorphics in ALI treatment in animal models, the mechanisms of these drugs are complex, and their curative effects are dubious. Therefore, types, doses, and mechanisms of senotherapies should be explored further using various ALI models.

## 9. Conclusion

The liver is an organ with great regenerative capacity. However, ALI is a challenging problem due to its etiological complexity and limited treatment options. Recent studies have shown that cellular senescence could be induced following ALI, even in young individuals; therefore, targeting cellular senescence is a potential ALI treatment.

Cellular senescence is a crucial cellular process in ALI that can either promote liver repair or exacerbate damage. However, current insights into hepatic senescence are insufficient. Therefore, further exploration of the heterogeneity of senescent cells, distinguishing between harmful and protective senescence, understanding the functions of senescent cells in ALI, and elucidating their detailed mechanisms is necessary. This will contribute to improved ALI treatment.
